# Incidence of Type 1 Diabetes in Children Aged Below 18 Years during 2013-2015 in Northwest Turkey

**DOI:** 10.4274/jcrpe.0025

**Published:** 2018-11-29

**Authors:** Şükran Poyrazoğlu, Rüveyde Bundak, Zehra Yavaş Abalı, Hasan Önal, Sevil Sarıkaya, Abdurrahman Akgün, Serpil Baş, Saygın Abalı, Abdullah Bereket, Erdal Eren, Ömer Tarım, Ayla Güven, Metin Yıldız, Derya Karaman Aksakal, Ayşegül Yüksel, Gülcan Seymen Karabulut, Şükrü Hatun, Tolga Özgen, Yaşar Cesur, Mehmet Azizoğlu, Emine Dilek, Filiz Tütüncüler, Esra Papatya Çakır, Bahar Özcabı, Olcay Evliyaoğlu, Songül Karadeniz, Fatma Dursun, Semih Bolu, İlknur Arslanoğlu, Gül Yeşiltepe Mutlu, Heves Kırmızıbekmez, Pınar İşgüven, Ala Üstyol, Erdal Adal, Ahmet Uçar, Nurcan Cebeci, Didem Bezen, Çiğdem Binay, Serap Semiz, Hüseyin Anıl Korkmaz, Nihal Memioğlu, Elif Sağsak, Havva Nur Peltek, Melek Yıldız, Teoman Akçay, Serap Turan, Tülay Güran, Zeynep Atay, Neşe Akcan, Filiz Çizmecioğlu, Oya Ercan, Aydilek Dağdeviren, Firdevs Baş, Halim İşsever, Feyza Darendeliler

**Affiliations:** 1 İstanbul University İstanbul Faculty of Medicine, Department of Pediatric Endocrinology, İstanbul, Turkey; 2 University of Kyrenia Faculty of Medicine, Department of Pediatric Endocrinology, Kyrenia, Turkish Republic of North Cyprus; 3 Kanuni Sultan Süleyman Training and Research Hospital, Clinic of Pediatric Endocrinology and Metabolic Disease, İstanbul, Turkey; 4 Marmara University Faculty of Medicine, Department of Pediatric Endocrinology, İstanbul, Turkey; 5 Kartal Training and Research Hospital, Clinic of Pediatric Endocrinology, İstanbul, Turkey; 6 Uludağ University Faculty of Medicine, Department of Pediatric Endocrinology, Bursa, Turkey; 7 Amasya University Faculty of Medicine, Department of Pediatric Endocrinology, Amasya, Turkey; 8 Göztepe Training and Research Hospital, Clinic of Pediatric Endocrinology, İstanbul, Turkey; 9 Kocaeli University Faculty of Medicine, Department of Pediatric Endocrinology, Kocaeli, Turkey; 10 Koç University Faculty of Medicine, Department of Pediatric Endocrinology, İstanbul, Turkey; 11 Bezmialem Vakıf University Faculty of Medicine, Department of Pediatric Endocrinology, İstanbul, Turkey; 12 Trakya University Faculty of Medicine, Department of Pediatric Endocrinology, Edirne, Turkey; 13 Bakırköy Dr. Sadi Konuk Training and Research Hospital, Clinic of Pediatric Endocrinology, İstanbul, Turkey; 14 Şevket Yılmaz Training and Research Hospital, Clinic of Pediatric Endocrinology, Bursa, Turkey; 15 İstanbul University Cerrahpaşa Faculty of Medicine, Department of Pediatric Endocrinology, İstanbul, Turkey; 16 Zeynep Kamil Women’s and Children’s Disease Training and Research Hospital, Clinic of Pediatric Endocrinology, İstanbul, Turkey; 17 Ümraniye Training and Research Hospital, Clinic of Pediatric Endocrinology, İstanbul, Turkey; 18 Düzce University Faculty of Medicine, Department of Pediatric Endocrinology, İstanbul, Turkey; 19 Sakarya University Faculty of Medicine, Department of Pediatric Endocrinology, Sakarya, Turkey; 20 Haseki Training and Research Hospital, Clinic of Pediatric Endocrinology, İstanbul, Turkey; 21 Medipol University Faculty of Medicine, Department of Pediatric Endocrinology, İstanbul, Turkey; 22 Şişli Etfal Training and Research Hospital, Clinic of Pediatric Endocrinology, İstanbul, Turkey; 23 Derince Training and Research Hospital, Clinic of Pediatric Endocrinology, Kocaeli, Turkey; 24 Okmeydanı Training and Research Hospital, Clinic of Pediatric Endocrinology, İstanbul, Turkey; 25 Çorlu State Hospital, Clinic of Pediatric Endocrinology, Tekirdağ, Turkey; 26 Acıbadem University Faculty of Medicine, Clinic of Pediatric Endocrinology, İstanbul, Turkey; 27 Balıkesir Atatürk State Hospital, Clinic of Pediatric Endocrinology, Balıkesir, Turkey; 28 American Hospital, Clinic of Pediatric Endocrinology, İstanbul, Turkey; 29 Gaziosmanpaşa Taksim Training and Research Hospital, Clinic of Pediatric Endocrinology, İstanbul, Turkey; 30 Edirne Sultan 1. Murat State Hospital, Clinic of Pediatric Endocrinology, Edirne, Turkey; 31 Medical Park Gaziosmanpaşa Hospital, Clinic of Pediatric Endocrinology, İstanbul, Turkey; 32 University of Near East Faculty of Medicine, Department of Pediatric Endocrinology, Nicosia, Turkish Republic of North Cyprus; 33 İstanbul University İstanbul Faculty of Medicine, Department of Public Health, İstanbul, Turkey

**Keywords:** Type 1 diabetes mellitus, childhood, incidence

## Abstract

**Objective:**

To assess the incidence of type 1 diabetes mellitus (T1DM) in children under 18 years of age in the northwest region of Turkey during 2013-2015.

**Methods:**

All newly diagnosed T1DM cases were recorded prospectively during 2013-2015. Total, as well as gender and age group specific (0-4, 5-9, 10-14 and 15-17 age) mean incidences per 100,000 per year were calculated.

**Results:**

There were 1,773 patients diagnosed during 2013-2015 (588 cases in 2013, 592 cases in 2014, 593 cases in 2015). Of these, 862 (48.6%) were girls and 911 (51.4%) were boys. The mean age at diagnosis was 9.2±4.2 years and it was not significantly different between girls (9.0±4.1 years) and boys (9.4±4.4 years) (p=0.052). The crude mean incidence was 8.99/100.000 confidence interval (CI) (95% CI: 8.58-9.42). Although mean incidence was similar between boys [8.98/100.000 (CI: 8.40 to 9.58)] and girls [9.01/100.000 (CI: 8.42 to 9.63)], there was male predominance in all groups except for 5-9 year age group. The standardized mean incidence was 9.02/100.000 according to the World Health Organization standard population. The mean incidence for the 0-4, 5-9, 10-14 and 15-17 age groups was 6.13, 11.68, 11.7 and 5.04/100.000 respectively. The incidence of T1DM was similar over the course of three years (p=0.95). A significant increase in the proportion of cases diagnosed was observed in the autumn-winter seasons.

**Conclusion:**

The northwest region of Turkey experienced an intermediate incidence of T1DM over the period of the study.

**What is already known on this topic?**

Incidence of type 1 diabetes mellitus (T1DM) peaked in the age groups 5-9 and 10-14 years. Diagnosis of T1DM showed a seasonal pattern peaking in autumn-winter.

**What this study adds?**

The incidence of type 1 diabetes mellitus in children and adolescents aged 0-17 years was 8.99/100,000 during 2013-2015 in the Northwestern region of Turkey and constant over the course of these 3 years.

## Introduction

Type 1 diabetes mellitus (T1DM) is a common, chronic disease in children and adolescents. In many populations, an increase in the incidence of T1DM in children has been observed (1,2,3). Studies have shown that the incidence of T1DM varies widely between and within countries (1,2,3,4). Seasonal variations in the presentation and gender differences in incidence of T1DM have been reported (5,6,7,8).

In Turkey, data on incidence and incidence trends of childhood T1DM are limited. Our aim was to determine the incidence of T1DM in children and adolescents (aged under 18 years) during the years 2013-2015 in the northwestern region of Turkey and to analyze the seasonal presentation pattern of T1DM in these children.

## Methods

Turkey is divided into seven geographical regions determined by topography and climate and defined by central government. The northwestern region, where this prospective study was conducted, is one of these regions. All children younger than 18 years of age, diagnosed as T1DM in pediatric endocrinology units in this region during 2013-2015 were included in the study. The pediatric endocrinology units’ locations were 11 university hospitals, 15 state hospitals and one private hospital.

The diagnosis of T1DM was made by the pediatric endocrinologist who took care of the child, according to the accepted criteria of the International Society for Pediatric and Adolescent Diabetes (9). The date of diagnosis of diabetes was accepted as the day of the first insulin injection.

In the Turkish health care system, all children aged 0-17 years with T1DM are referred to a pediatric endocrinology department for treatment. Over the three year period (2013-2015), data on all hospitalized or referred new cases in the institutions in the Northwestern region of the country were reported to our team on a special form containing information about the patient’s personal identification number, sex, date of birth, date of diagnosis and some clinical and laboratory data. All forms were sent monthly to one investigator (SP) for data collection and verification. We excluded children with type 2 diabetes mellitus, neonatal diabetes, maturity onset diabetes of youth, transient hyperglycemia, and diabetes caused by other conditions (chemotherapy, cystic fibrosis, etc).

### Statistical Analysis

Incidence of T1DM was calculated using the numbers of patients reported for each year by age (0-4, 5-9, 10-14 and 15-17 years aged) and gender groups (girls and boys). Annual numbers for the age groups in the geographical area were used as denominators, and incidence (per 100,000 per year) was calculated with 95% CIs, assuming a Poisson distribution. The annual population sizes were obtained from the Turkish census data of 2013-2015 from the address-based population registration system of the Turkish Statistical Institute. For comparison with data from other countries, the incidence was standardized by the direct method according to the age distribution of the world population (10).

The percentage of patients diagnosed during each calendar month was calculated in age groups for both sexes and then compared, to identify any seasonal variation in diagnosis of T1DM.

In order to assess the significance of the differences between the groups, normality of variables was tested by Kolmogorov Smirnov test; Mann-Whitney U and chi-square tests were used. Results are reported as means ± SD. Two-tailed p values were calculated. Statistical significance was accepted as p<0.05.

## Results

A total of 1773 cases were identified over the three year period (588 cases in 2013, 592 cases in 2014, 593 cases in 2015). Of these, 862 (48.6%) were girls and 911 (51.4%) were boys, giving a male to female ratio of 1.05:1. The mean age at diagnosis was 9.2±4.2 years and showed no sex difference (9.0±4.1 years in the girls and 9.4±4.4 years in the boys, p=0.052). Table 1 shows mean ages and distribution of the patients by age groups over the three year period. The proportion of newly diagnosed T1DM cases was highest among children aged 5-9 years (35.9%), followed by the age groups 10-14 years (35.3%), 0-4 years (19.1%) and 15-17 years (9.6%).

The crude mean annual incidence in children aged 0-17 years over this period was 8.99 per 100,000 [95% confidence interval (CI): 8.58 to 9.42]. The standardized mean incidence was 9.02 per 100,000 according to the World Health Organization (WHO) standard population.

There was no significant difference between the mean annual incidence figures for boys [8.98/100.000 (CI: 8.40 to 9.58)] and girls [9.01/100.000 (CI: 8.42 to 9.63)] during the study period (p=0.95) (Table 2). The mean annual incidence for the 0-4 year age group was 6.13/100.000. Incidence increased significantly with age, reaching a peak in the age groups 5-9 and 10-14 years. It was 11.68/100.000 for the 5-9 year age group and 11.7/100.000 for the 10-14 year age group and subsequently the incidence declined at age 15-17 years. The lowest incidence was seen in the age group 15-17 years (5.04/100,000) (Table 2). The incidence of the age group 0-14 years was 9.82/100,000 (95% CI: 9.34 to 10.31). Male predominance was seen in all groups except for the 5-9 years age group (Table 2). The incidence of T1DM was similar over the course of the three years (Table 2, p=0.95).

**Figure 1:**
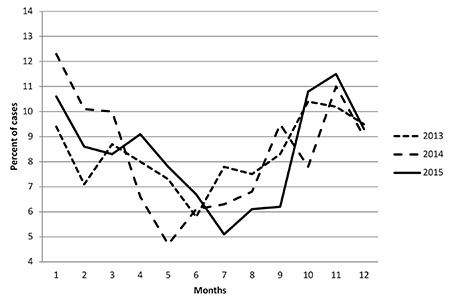
Distribution of age of onset of type 1 diabetes according to months of the year

A significant increase in proportion of diagnosis of T1DM was observed in the autumn-winter seasons (Figure 1). It was similar over the three year period in all age and gender groups.

## Discussion

In this study we investigated the incidence of T1DM in children residing in the Northwestern region of Turkey, our results demonstrate that the incidence of T1DM here is intermediate (8.99/100,000) in the pediatric age group (4). In Turkey, there are only a few reports on the epidemiology of T1DM in children and most of them focused on children below 15 years of age (11,12,13,14). Our study is one of the few population-based reports presenting the incidence of T1DM among children in Turkey.

Considerable differences in incidence rates for T1DM have been reported from different countries, and even within the same country (1,2,3,4). Recently, in 2013, a nationwide incidence of T1DM among Turkish children was reported and this study covered T1DM incidence in Turkey divided 5 geographic regions (14) with notable differences in incidence across the five regions. The northwestern region is a developing part of Turkey and there have been big changes in the economy, urbanization and lifestyles in recent decades in this region. A slightly higher incidence (10.1/100,000 per year) was reported in the all western part of Turkey in this nationwide study and the results were consistent with our data (9.01/100,000 per year). However, the methodology for case ascertainment used in our study is different from the nationwide study. The nationwide study used data from the universal health insurance system about prescriptions for essential medicines for diabetics for the calculation of incidence. In our study the data were collected prospectively from T1DM patient data from pediatric endocrinology units in the region.

We cannot detect the incidence trends from our study due to the short period covered and the lack of epidemiological data before our study in children younger than 18 years in Turkey. Our neighbour countries reported intermediate rates for incidence, similar to our results (2,3,15) and reported an increase in incidence of T1DM over time. Although our observation period was very short, incidence was quite stable over the three year period. Although the global increase in the incidence of T1DM is widely recognized in recent decades, some studies in populations with a higher incidence of T1DM have demonstrated that the increase in the incidence slowed down in the last decade (16,17,18). In the EuroDiab study, it was reported that between 2001 and 2009 the increase in T1DM incidence was significantly different in regions within Europe and the highest increase occurred in Central Eastern European countries while Finland, other Nordic countries and the Czech Republic showed a much lower increase or a stabilization in the incidence of T1DM (16,17,19,20,21). An average relative increase of 3-4% per year has been reported worldwide (22). Environmental factors are thought as the most likely reason for this increase in incidence (3,20,23). For this reason, evaluation of incidence in different regions is important and warranted.

In our cohort, although the mean annual incidence for boys and girls was similar, a male predominance was seen in all age groups except for the age group 5-9 years. The female predominance in the 5-9 years age group could be due to the earlier onset of puberty in girls than in boys. Gender differences in T1DM have been identified in many studies (4,6,24,25,26,27). Overall, high incidence countries tend to have a slight male predominance and low incidence countries a female predominance (4,24,25,26,27). Karvonen et al (4) found that 88% of low incidence populations were predominantly girls and patients in high incidence populations were more likely to be predominantly boys. In Sardinia, a very high incidence area, a male predominance is reported in the 0-14 year age group (26). The Danish Study Group of Diabetes in Childhood reported male predominance in their population (24). However, no significant difference in T1DM incidence between boys and girls was observed in Shanghai or in Kuwait (25,27).

Age differences in T1DM incidence have also been observed in previous studies (2,15,27,28,29). The incidence in our cohort increased with age in both sexes and was highest in the 5-14 year age group. This was followed by a decrease in the 15-17 year age group. Incidence was similar in children aged 5-9 and 10-14 years in our study. The youngest age group (0-4 years) had a lower incidence as compared with older children (5-14 years). This difference in incidence by age groups has also been shown in other counties. The WHO analyzed standardized incidence data on T1DM in the Multinational Project (DIAMOND) in 112 centres from 57 countries during 1990-1999. The DIAMOND study showed that 5-9 years old children had a higher risk of developing T1DM compared with 0-4 year old children (2). Some countries reported a high incidence in the 5-9 years old group, but others found the highest incidence in children aged 10-14 years (2,15,27,28,29).

Our cohort showed a significant seasonal variation in diagnosis of T1DM. More cases were diagnosed during autumn and winter months, which are the cooler seasons in the Northwest region. This seasonality of diagnosis of T1DM was identified in both sexes and in all age groups and thus seems to be a robust finding. Although Turkey is situated in the Mediterranean geographical location, the diverse regions have different climates because of irregular topography. To evaluate the impact of weather on incidence of TIDM in Turkey, each region should be evaluated separately. Similar to our results some countries show significant seasonality in diagnosis for all age groups, with higher incidence rates in the winter period (5,7,8). Some countries reported no seasonality in all age groups or absent in some age groups (5,8,30,31,32). Different interpretations have been suggested for this seasonal pattern in diagnosis of T1DM, including physical activity, stress, viral infections and vitamin D synthesis during different seasons (5,7,8,33). The DIAMOND group reported seasonality in T1DM incidence with winter or summer peaks in 40% of all participating centers depending on the geographic position of the country (2). It was shown that in Japan there was a bimodal pattern in the diagnosis of T1DM, that is common in April/May and in December with no seasonal pattern of incidence among preschool children (6).

### Study Limitation

The limitation of our study was the short duration of the registry. It would be important to continue monitoring incidence of T1DM in the same region and evaluate trends.

## Conclusion

To conclude, this is the first paper that analyzes the time-related trends in the incidence of T1DM in Turkish children aged from 0 to 17 years in the Northwest region of Turkey. The results showed an intermediate incidence of T1DM and a similar mean annual incidence between boys and girls. Considering the increasing incidence of T1DM worldwide, we suggest that it would be important to follow trends in incidence in the next few years in this same region to determine the possible triggering factors and also to develop preventive strategies.

## Figures and Tables

**Table 1:**
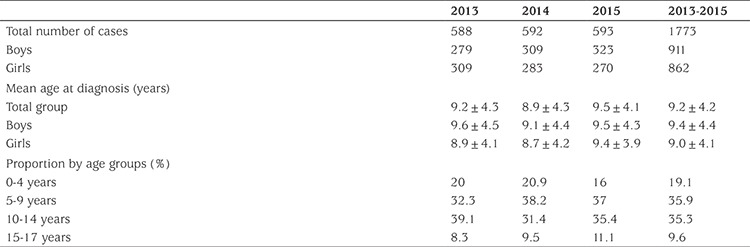
Mean ages at diagnosis and distribution by age groups of newly diagnosed type 1 diabetes mellitus cases over the three year period

**Table 2:**
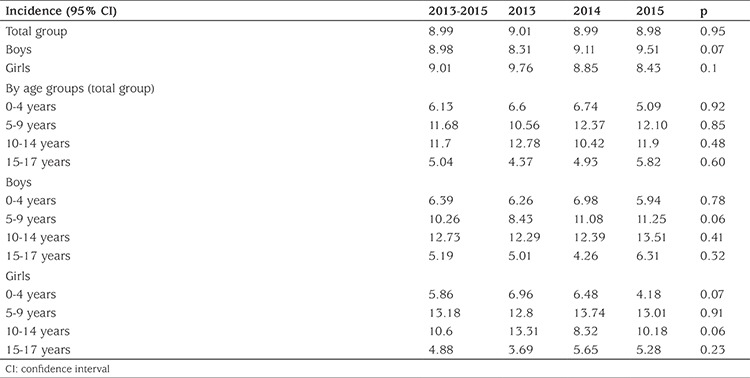
Incidence of type 1 diabetes mellitus over the three year period
